# The roles of metallothioneins in carcinogenesis

**DOI:** 10.1186/s13045-018-0645-x

**Published:** 2018-08-23

**Authors:** Manfei Si, Jinghe Lang

**Affiliations:** Department of Obstetrics and Gynecology, Peking Union Medical College Hospital, Peking Union Medical College, Chinese Academy of Medical Sciences, No. 1 Shuaifuyuan, Dongcheng District, Beijing, 100730 China

**Keywords:** Metallothionein, Metal homeostasis, Cancer, Carcinogenesis, Biomarker

## Abstract

Metallothioneins (MTs) are small cysteine-rich proteins that play important roles in metal homeostasis and protection against heavy metal toxicity, DNA damage, and oxidative stress. In humans, MTs have four main isoforms (MT1, MT2, MT3, and MT4) that are encoded by genes located on chromosome 16q13. MT1 comprises eight known functional (sub)isoforms (MT1A, MT1B, MT1E, MT1F, MT1G, MT1H, MT1M, and MT1X). Emerging evidence shows that MTs play a pivotal role in tumor formation, progression, and drug resistance. However, the expression of MTs is not universal in all human tumors and may depend on the type and differentiation status of tumors, as well as other environmental stimuli or gene mutations. More importantly, the differential expression of particular MT isoforms can be utilized for tumor diagnosis and therapy. This review summarizes the recent knowledge on the functions and mechanisms of MTs in carcinogenesis and describes the differential expression and regulation of MT isoforms in various malignant tumors. The roles of MTs in tumor growth, differentiation, angiogenesis, metastasis, microenvironment remodeling, immune escape, and drug resistance are also discussed. Finally, this review highlights the potential of MTs as biomarkers for cancer diagnosis and prognosis and introduces some current applications of targeting MT isoforms in cancer therapy. The knowledge on the MTs may provide new insights for treating cancer and bring hope for the elimination of cancer.

## Background

Metallothioneins (MTs) are a family of low molecular weight (ranging from 6 to 7 kDa), cysteine-rich cytosolic proteins that play a vital role in metal ion homeostasis and detoxification [1, 2]. MT was first isolated by Margoshes and Vallee from horse kidney cortex as a low molecular weight protein containing cadmium in 1957 [3]. MTs are involved in metalloregulatory processes by binding to heavy metals through the thiol group of their cysteine residues. MTs have a high affinity for heavy metals, which means that they can bind to xenobiotic heavy metals to provide protection against metal toxicity, especially cadmium toxicity [4]. When MTs bind to physiological heavy metals, such as zinc and copper, they can participate in regulating cell growth and proliferation and protecting the body against oxidative stress [5]. Recently, many studies have shown that MT expression varies in different tumors, suggesting that MTs may play a vital role in carcinogenesis [6–10]. The elucidation of possible functions and mechanisms of MTs in tumor progression may provide potential promising markers for cancer. This review was conducted to summarize the latest data on the role of MTs in carcinogenesis and to provide diagnostic or therapeutic information to help oncologists in their clinical decision-making.

### Structure and classification

MTs are highly conserved, low molecular weight proteins that are present in a broad range of taxonomic groups and display a high level of sequence heterogeneity, which results in varying molecular weights and number and distribution of cysteine residues [1, 11]. Mammalian MTs constitute a superfamily of nonenzymatic polypeptides of 61–68 amino acids, characterized by high cysteine content (30%), lack of aromatic amino acids, and few or no histidine residues but with abundant thiol groups to bind to heavy metals [9, 11, 12].

In humans, MTs are encoded by a family of genes located on chromosome 16q13 and include at least 11 functional members: MT1 (MT1A, MT1B, MT1E, MT1F, MT1G, MT1H, MT1M, and MT1X; MT1C, MT1D, MT1I, MT1J, and MT1L are pseudogenes that cannot encode MT proteins), MT2 (also known as MT2A), MT3, and MT4 [13, 14]. A summary of MT genes, isoforms, and location is shown in Table 1.

**Table 1:** Genetic information related to functional MT isoforms obtained from the National Center of Biotechnology Information (NCBI)

MT isoforms	Gene ID	Location	GenBank accession number	References
MT1A	4489	Chromosome 16, NC_000016.10 (56638666..56640087)	NM_005946.2	[170, 171]
MT1B	4490	Chromosome 16, NC_000016.10 (56651899..56653204)	NM_005947.2	[172, 173]
MT1E	4493	Chromosome 16, NC_000016.10 (56625673..56627112)	NM_175617.3	[143, 172]
MT1F	4494	Chromosome 16, NC_000016.10 (56657943..56659303)	NM_005949.3	[131, 174]
MT1G	4495	Chromosome 16, NC_000016.10 (56666735..56668065, complement)	NM_005950.2	[172, 175]
MT1H	4496	Chromosome 16, NC_000016.10 (56669814..56671129)	NM_005951.2	[8, 176]
MT1M	4499	Chromosome 16, NC_000016.10 (56632622..56633986)	NM_176870.2	[142, 172]
MT1X	4501	Chromosome 16, NC_000016.10 (56682470..56684196)	NM_005952.3	[176, 177]
MT2A	4502	Chromosome 16, NC_000016.10 (56608566..56609497)	NM_005953.4	[178, 179]
MT3	4504	Chromosome 16, NC_000016.10 (56589355..56591088)	NM_005954.3	[31, 180]
MT4	84560	Chromosome 16, NC_000016.10 (56565049..56568957)	NM_032935.2	[32, 181]

### Roles of MTs in cancer

**Fig. 1:**
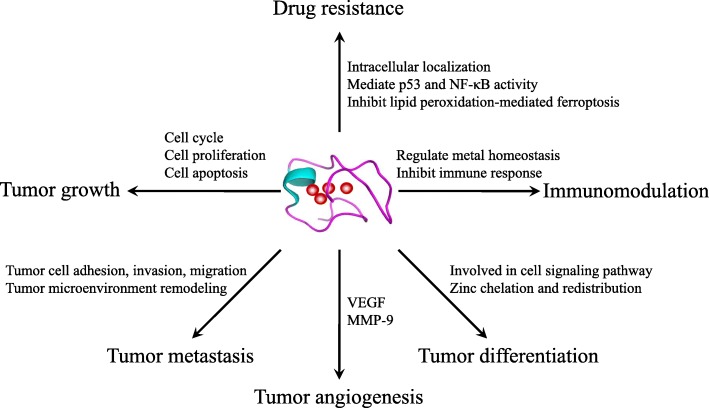
Roles of MTs in carcinogenesis

The well-known biological functions of MTs are related to their high affinity for heavy metals. MTs can control cellular homeostasis of zinc/copper, which is essential for cell proliferation and differentiation, and act as antioxidants to protect cells against free radicals and oxidative stress generated by mutagens, antineoplastic drugs, and radiation [15, 16]. MTs can also bind to cadmium, mercury, platinum, or other similar heavy metals to protect cells and tissues against heavy metal toxicity [4]. In addition, MTs play a protective role against DNA damage and apoptosis [17–19]. Accumulating evidence indicates that MTs play important roles in carcinogenesis and cancer therapy. MTs participate in the process of carcinogenesis and play critical roles in tumor growth, progression, metastasis, and drug resistance (Fig. 1). To provide a comprehensive insight into the complicated relation between MTs and cancer, we summarized the dysregulated expression and functions of MT isoforms in various tumor tissues in Table 2.

**Table 2:** Overview of the dysregulated expression and functions of MT isoforms in cancer

Cancer type	MT isoforms	Expression	Functions	References
Acute nonlymphoblastic leukemia	MT	Positive	Resistance-related proteins	[182]
Acute myeloid leukemia	MT1G, MT1A	Positive	Inversely correlated with PU.1 expression	[183]
	MT3	Down	Promoter hypermethylation	[154]
Adenoid cystic carcinomas of the salivary glands	MT	Up	Myoepithelial differentiation	[184]
Basal cell carcinoma	MT	Up	Infiltrative growth	[185]
	MT1, MT2	Up	Promote proliferation: Ki-67 antigen expression	[43]
	MT3	Down	Possibly based on DNA methylation	[186]
	MT3	Low to moderate expression	Carcinogenesis	[187]
Bladder carcinoma	MT	Up	Drug resistance	[188]
	MT2	Up	Cisplatin resistance	[99]
	MT3	Up	Carcinogenesis and increase tumor grade	[189]
	MT1X	Up	Correlated with tumor grade	[190]
	MT	Up	Poor survival and cisplatin resistance	[23]
Breast cancer	MT3	Up	Poor prognosis	[130]
	MT2A	Up	Increase invasiveness	[191]
	MT1E	Present in estrogen receptor (ER)-negative breast cancer	Myoepithelial differentiation and tumor invasiveness	[191]
	MT2A	Up	Modulate cell cycle via the ATM/Chk2/cdc25A signaling pathway	[39]
	MT2A	Up	Upregulation of matrix metalloproteinase (MMP)-9; enhance cell invasion and migration	[82]
	MT3	Up	Increase invasiveness	[83]
Cholangiocarcinoma	MT	Partly positive	Poor prognosis	[128]
Colonic cancer	MT	Up	Promote proliferation	[36]
	MT1F	Down	Loss of heterozygosity	[52]
	MT1G, MT1X, MT2A	Down	Associated with the depth of tumor invasion, lymph node metastasis, and tumor stage	[52]
Colorectal cancer	MT2A, MT1B, MT1F, MT1G, MT1H, MT1B, MT1F, MT1G, MT1H, MT2A	Down	Poor clinical outcome	[192]
	MT1E, MT1F, MT1G, MT1H, MT1M, MT1X MT2A	Down	Epigenetic mechanisms	[6]
	MT2A	Up	Interact with Fas-associated death domain (FADD) in NF-κB pathway to promote cell proliferation	[49]
	MT1G	Down	Colorectal cancer cell differentiation	[67]
Ductal breast cancer	MT1E	High in ER-negative cancer tissues	Mediate effector genes downstream of ER	[193]
	MT1F	Up	Influence histological differentiation	[58]
	MT2A	Up	Cell proliferation	[41]
	MT2A	Up	Chemoresistance (doxorubicin)	[59]
	MT1, MT2	Up	Increased proliferative potential	[20]
	MT3	Down	Epigenetic changes	[88]
Endometrial carcinoma	MT1, MT2	Up	Modify p53 expression	[141]
	MT1E	Down	Promoter hypermethylation	[143]
Esophageal adenocarcinoma	MT3	Down	DNA methylation	[152]
Esophageal squamous cell carcinoma	MT	Up	Chemoresistance to cisplatin; poor prognosis	[194]
	MT3	Down	DNA methylation	[153]
	MT1G	Down	Gene methylation	[145]
	MT1G	Down	Promoter hypermethylation	[146]
	MT1M	Down	DNA methylation; correlated with smoking duration	[195]
Gallbladder carcinoma	MT	Up	Histological dedifferentiation	[63]
Gastric carcinoma	MT3	Down	Hypermethylation	[151]
	MT1G	Up	Cisplatin resistance	[196]
	MT1X	Up	Irinotecan resistance	[103]
	MT	Up	Poor survival and high recurrence rate	[81]
	MT2A	Down	Inhibit the activation of the NF-κB pathway	[135]
	MT2A	Down	Be a potential target of miR-23a	[197]
	MT1D (MTM)	Down	Enhance migration and invasion	[198]
	MT1M, MT1JP	Down	Associated with tumor diameter, differentiation, lymphatic metastasis, distal metastasis, invasion, and tumor node metastasis (TNM) stage	[199]
Glioma	MT1E	In proportion to the motility of glioma cell	Enhance tumor proliferation, invasion, and migration through regulation of activation and expression of MMPs	[84, 85]
Hepatoblastoma	MT1G	Down	Promoter hypermethylation	[147]
Hepatocellular carcinoma	MT1F	Down	Cell growth	[200]
	MT1G	Down	Allelic loss on chromosome 16q12.1-q23.1	[155]
	MT1, MT2A	Down	Transcriptional repression: dephosphorylation of the transcription factor CCAAT/enhancer-binding protein (C/EBP) α through phosphatidylinositol 3-kinase (PI3K)/AKT signaling pathway	[25]
	MT1X, MT2A	Down	Malignant transformation of hepatocytes; local invasion; hepatitis B virus infection	[201]
	MT1G	Down	Tumor suppressor gene; promoter hypermethylation	[148]
	MT1M	Down	Increase NF-κB activity	[142]
	MT1, MT2	Down	Promoter hypermethylation and transcriptional repression; prognostic marker	[133]
	MT1M, MT1G	Down	Promoter methylation	[136]
	MT1M	Down	Poor prognosis	[134]
	MT1, MT2	Down	Associated with the disruption of circadian clock genes	[202]
	MT1H	Down	Regulate the Wnt/β-catenin signaling pathway	[8]
	MT1M	Down	Inhibit tumorigenesis	[203]
Intrahepatic cholangiocarcinoma	MT1A, MT1E, MT1F, MT1G, MT1H, MT1IP, MT1X	Down	Hypermethylation	[204]
Lung cancer	MT1A, MT2A, MT1E, MT1G	Down	Gene methylation	[205]
Large cell lung cancer	MT1F, MT1G, MT1M, MT1X	Up	Poor prognosis	[206]
Melanoma	MT	Up	Poor prognosis	[24, 125]
	MT1E	Down	DNA methylation	[144]
	MT1, MT2	Up	Intratumoural macrophage infiltration to defect host immune response and metastasis formation	[77]
Nasopharyngeal cancer	MT	Up	Cell proliferation	[21]
Non-small cell lung cancer	MT	Up	Tumor cell proliferation and short survival	[122]
	MT1H	Up	Drug resistance (cisplatin)	[207]
	MT1, MT2	Up	Promote proliferation: expressions of Ki-67 and minichromosome maintenance protein-2 (MCM-2) (positive correlation)	[40]
	MT3	Up	Pathogenesis	[208]
	MT1B, MT1F, MT1G, MT1H, MT1X	Up	Pathogenesis	[131]
	MT1F, MT2A	Up	Poor outcome	[131]
	MT1E	Down	Cell differentiation	[131]
Oral squamous cell carcinoma	MT	Up	Poor prognosis	[127]
	MT1A, MT1X, MT3, MT4	Down	Possible markers for oral carcinogenesis	[209]
	MT1G	Down	Poor survival	[209]
	MT1F	Up	Associated with tobacco use	[209]
Osteosarcoma	MT1E, MT1H, MT1X, MT2A, MT1B, MT1G, MT1L	Up	Drug resistance	[210]
Ovarian cancer	MT1, MT2	Up	Mutant p53; histological grade	[22]
	MT2A	Up	Inhibit cell death	[211]
	MT1L, MT1X, MT2A	Up	Low malignant potential or early cancer onset	[212]
Pancreatic carcinoma	MT	Partly positive	Metastasis, poor prognosis, and poor histological grade	[62]
Papillary thyroid carcinoma	MT1G	Down	Promoter hypermethylation	[149]
	MT1E, MT1G, MT1X, MT2A	Down	Promoter methylation and transcriptional repression	[7]
	MT1G	Down	Modulate the activity of the PI3K/AKT and Rb/E2F pathways	[89]
Prostate cancer	MT3	Highly variable increase	Control prostate epithelial cell growth	[213]
	MT1X	Down	Advanced prostate cancer	[214]
	MT2A	Up	Inhibit cell death	[211]
	MT3	Up	Inhibit cell growth and increase drug resistance	[53]
	MT1G	Down	Promoter hypermethylation	[150]
	MT1F, MT1M	Down	Associated with perineural invasion	[215]
	MT1H	Down	Enhance the histone methyltransferase activity of euchromatin histone methyltransferase 1 (EHMT1)	[26]
	MT2A	Down	Single nucleotide polymorphism (SNP); metal accumulation	[14]
	MT3	Depend on cell type	Increase cell proliferation, invasion, and tumorigenic activities	[104]
	MT1E	Down	DNA methylation	[10]
Renal cell cancer	MT	Up	Tumor grade	[64]
	MT2A	Up	Stimulate cellular proliferation	[216]
	MT1A, MT1G	Down	Growth arrest and induction of apoptosis	[216]
	MT1A, MT1E, MT1G, MT1H, MT1L	Down	Tumorigenesis	[217]
	MT1H, MT1G, MT2A	Down	Promoter methylation	[45]
Salivary gland adenocarcinoma	MT	Up	High immunoreactivity and microenvironment remodeling	[90]
Serous ovarian cancer	MT	Up	Diagnosis of malignancy and worse prognosis	[218]
Small cell lung cancer	MT	45% positive	P53 expression and short-term survival	[121]
Soft tissue sarcoma	MT	Up	Ki-67 expression, grade of malignancy, and prognostic appraisal	[44]
	MT2A, MT1X, MT1F, MT1H	Up	Metastasis	[219]
Squamous cell carcinoma of the tongue	MT	Positive	Delay cells entering apoptosis	[220]
	MT	Positive	Correlated with depth of invasion, vascular invasion, and lymph node metastasis	[221]
Testicular cancer	MT	Up	Chemoresistance	[222]
	MT	Up	Early diagnosis	[115]
Transitional cell carcinoma of the bladder	MT	Up	Poor survival	[126]

#### Expression of MT isoforms in various types of cancers

Numerous studies have demonstrated that changes in MT expression are associated with the process of carcinogenesis and cancer progression. However, the expression of MTs is not universal in all human cancers. Previous studies have shown that MT expression is upregulated in breast cancer, nasopharyngeal cancer, ovarian cancer, urinary bladder cancer, and melanoma [20–24], while in other cancers, such as hepatocellular carcinoma, prostate cancer, and papillary thyroid carcinoma, MT expression is downregulated [7, 25, 26]. Theocharis et al. also observed that among lung cancer subtypes, MT expression was prominent in squamous cell lung carcinoma and adenocarcinoma but absent in small cell lung cancer [27]. The differential expression of MTs depends on the type and differentiation status of tumors, as well as other environmental stimuli and/or gene mutations [17, 28, 29]. Nevertheless, a better understanding of the changes in the expression of particular MT isoforms in various cancers can help identify specific therapeutic targets and reverse tumor progression.

In humans, MTs have four main isoforms: MT1, MT2, MT3, and MT4. Of these four isoforms, MT1 and MT2 are ubiquitously expressed in various tissues, while MT3 and MT4 are minor isoforms with restricted expression in specialized cells and tissues, such as the brain, reproductive organs, and stratified squamous epithelium [7, 17]. MT3 was first purified and characterized as a growth inhibitory factor (GIF) in the human brain [30] and was later designated as a third member of the MT family [31]. MT4 was discovered in the stratified squamous epithelium in the skin, esophagus, and tongue [32]. Furthermore, MT1 and MT2 are basally expressed and highly induced by a variety of stimuli including metals, hormones, cytokines, growth factors, oxidants, stress, and irradiation, while MT3 and MT4 are constitutively expressed despite signal changes in vitro or in vivo [33].

**Fig. 2:**
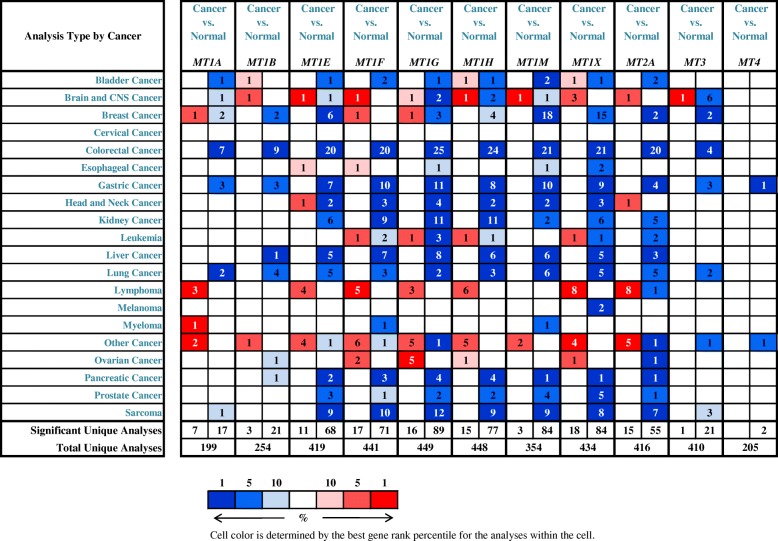
Transcript levels of MT isoforms in different types of cancers. This figure was generated from Oncomine, indicating the numbers of datasets with statistically significant MT mRNA upregulation (red) or downregulation (blue) (different types of cancer vs. corresponding normal tissues) (threshold setting: *p* value, 0.01; fold change, 2; gene rank, top 10%). The number in the colored cell represents the number of datasets meeting the threshold

Krizkova et al. have provided a comprehensive summary of the expression and regulation of individual MT isoforms in various types of malignancies [34]. In Fig. 2, the transcript levels of MT isoforms in cancers are compared with those in normal samples by using Oncomine databases (threshold setting: *p* value, 0.01; fold change, 2; gene rank, top 10%). The figure shows that the mRNA levels of MT isoforms are significantly up/downregulated in various types of cancers (Fig. 2). These data clearly indicate that MT isoforms can be targeted to treat cancer and enhance the efficiency of anticancer therapy due to their important roles and altered expression in various cancers. Therefore, knowledge on the expression of MT isoforms could be fully utilized for tumor diagnosis and anticancer therapy.

#### Tumorigenesis

Tumorigenesis, also called carcinogenesis or oncogenesis, refers to the formation of a tumor in which normal cells are transformed into cancer cells. Any changes at the cellular, genetic, and epigenetic levels that disrupt the balance between proliferation and programmed cell death, in the form of apoptosis, such as DNA mutations and epimutations, can contribute to the development of cancer. MTs have been shown to play an important role in carcinogenesis. To better understand the roles of MTs in cancer, the National Cancer Institute held a workshop that focused on three topics: the role of zinc in tumor cell pathobiology, the role of MTs in metal carcinogenesis, and the role of MTs in tumor cells and potential in cancer chemotherapy [35]. Since then, a large number of studies have been conducted to investigate the roles of MTs in carcinogenesis.

#### Tumor growth

The tumor growth-promoting effects of MTs involve the following potential mechanisms. Nagel et al. demonstrated that cytoplasmic MTs reached a maximum level during the G1/S cell cycle transition, a period when cells prepare for DNA synthesis, thereby demonstrating a physiological role of MTs in tumor cell proliferation [36]. Studies have shown that zinc is required for G1/S phase transition [37, 38]. Thus, it can be hypothesized that MTs regulate the supply of zinc for proteins and the activity of zinc-dependent transcription factors to modulate tumor cell growth and proliferation. Later, Lim et al. observed that the downregulation of MT2A expression in breast cancer cells could induce cell cycle arrest at the G1 phase to inhibit cancer cell growth, and the underlying molecular mechanisms involved the regulation of cell cycle-related genes including ataxia telangiectasia mutated (ATM) and cell division cycle 25A (cdc25A) [39].

MTs also participate in cell proliferation. Ki-67 is one of the most sensitive markers of cell proliferation. Werynska and colleagues have shown a positive correlation between the expression of MT1/2 and that of the proliferation markers Ki-67 and minichromosome maintenance protein 2 (MCM-2) in non-small cell lung cancer [40]. A similar positive correlation between MTs and Ki-67 expression had also been confirmed in breast cancer [20, 41], nasopharyngeal carcinoma [21], large intestine adenocarcinoma [42], basal cell carcinoma [43], and soft tissue sarcomas such as malignant fibrous histiocytoma, liposarcoma, and synovial sarcoma [44].

MTs have also been shown to inhibit apoptosis [19]. MTs can act as zinc donors for transcription factors such as hypoxia-inducible factor-1α (HIF-1α) and tumor suppressors such as P53 to influence cell growth [17, 45]. P53 is a zinc-binding transcription factor that can inhibit cell cycle progression and induce apoptosis in response to DNA damage. MTs can remove zinc ions from P53 protein molecules, thus leading to the changes in the spatial structure of P53, leading to its inactivation and thus result in uncontrolled cell proliferation [20, 46]. Moreover, MTs have been demonstrated to interact with nuclear factor-κB (NF-κB) and mediate its antiapoptotic effects [47], probably because zinc is an essential component for the DNA-binding function of NF-κB [48]. Marikar et al. revealed a new phenomenon that the interaction of the phosphorylated Fas-associated death domain (FADD) with MT2A was involved in the increase in cell proliferation and inhibition of cell apoptosis in colorectal cancer via the NF-κB pathway [49]. MT expression can protect cells from a variety of apoptotic stimuli, including oxidative stress, heavy metals, and chemotherapeutic agents (doxorubicin, etoposide, etc.) [18, 50, 51].

Intriguingly, in vitro experiments have confirmed that MT1F transfection into colon cancer cells could decrease cell proliferation and colony formation and increase cell apoptosis rates to inhibit cell growth. The authors also obtained similar results with in vivo experiments in which compared with those in empty vector-expressing mice, the tumor growth rate and average tumor size and weight were reduced in MT1F-expressing mice. These results highlighted MT1F as a tumor suppressor that can inhibit tumor growth in vivo [52]. Similarly, low expression and tumor suppressor activity of MT1H were identified in prostate cancer. In detail, the induced expression of MT1H reduced the colony formation and decreased the entry of prostate cells into the S and M phases to suppress cell growth [26]. A similar suppressive role of MT1H was also reported in hepatocellular carcinoma [8]. Dutta et al. demonstrated that the stable transfection of PC-3 cells to overexpress the MT3 gene significantly reduced the cell growth relative to both nontransfected PC-3 cells and blank vector-transfected control cells [53].

Altogether, these findings indicate that MTs may contribute to tumor growth by regulating cell cycle arrest, cell proliferation, and apoptosis. However, whether MTs play an oncogenic or tumor-suppressive role depends on their isoforms and the type of tumors.

#### Tumor differentiation

Cellular differentiation is the process by which a cell changes from one type to another. The “grade” of histological differentiation or the grade of malignancy is used as a measure of cancer progression and includes the ability to form glandular structures, cellular polymorphism, and evident mitotic activity. Many studies have reported that MTs participate in cell differentiation. Aikins and fellow researchers investigated the influence of extremely low-frequency electromagnetic fields (ELFEMFs) on zinc-MT3 interactions during the neural differentiation of human bone marrow-derived mesenchymal cells. Their study found that during this interaction, MT3 expression was downregulated, and the formation of zinc-MT3 complexes was enhanced to maintain zinc homeostasis. A new homeostatic regulatory mechanism was thus discovered, which involved the zinc-MT3 complex and other MT3-interacting proteins to drive neural differentiation, thereby highlighting the potential diagnostic and clinical applications for MT3 in neurodegenerative diseases [54]. Moreover, Wu et al. demonstrated MTs as negative regulators for interleukin (IL)-27-induced type 1 regulatory T cell differentiation [55]. Hirako et al. uncovered that the overexpression of MT1G could inhibit the differentiation of all-trans retinoic acid (ATRA)-induced NB4 acute promyelocytic leukemia cells [56]. MT2A expression was reported to influence the osteosarcoma cell differentiation toward the osteogenic lineage. In other words, MT2A overexpression could the enhance cell differentiation [57]. Additionally, numerous studies have revealed a relationship between MT expression and tumor differentiation. Jin et al. found that the expression of MT1F and MT2A in histological grade 3 breast cancer was significantly higher than that in histological grades 1 and 2 in breast cancer [41, 58]. Similar results were recently demonstrated by other authors [20, 59–61]. The relationship between MT expression and tumor histological grade was also demonstrated in pancreatic ductal carcinoma [62], gallbladder carcinoma [63], renal cancer [64], ovarian adenocarcinoma [22, 65], and endometrial carcinoma [66]. All of the mentioned studies demonstrated a strong positive correlation between MT expression and tumor grade, showing that MT expression was enhanced with increasing tumor grade. To investigate the role of MT1G in the differentiation of colorectal cancer cells, Arriaga et al. transfected MT1G-myc expression plasmids into H29 cells, which stably overexpress MT1G. The authors uncovered that MT1G was involved in the process of tumor cell differentiation mainly through the Notch signaling pathway and labile zinc chelation and redistribution [67].

Cell differentiation is necessary for normal development. Undifferentiated and poorly differentiated cells have a high likelihood to form tumors. As mentioned above, MTs have been reported to participate in cell differentiation and have been found to be positively correlated with histological tumor grade. These findings may provide a new approach for treating cancers in which tumor cells are prompted to differentiate into more mature cells by using MT-related pharmacological agents.

#### Tumor angiogenesis

The formation of new blood vessels is a required step for tumorigenesis because tumors need a network of blood vessels to obtain sufficient oxygen and essential nutrients for their growth, progression, and metastasis [68]. Several studies have demonstrated that MTs play an important role in tumor angiogenesis. Miyashita and Sato showed that MT1 was expressed in vascular endothelial cells (ECs) at the site of angiogenesis, and the downregulation of MT1 expression inECs resulted in the inhibition of cell proliferation, migration, and angiogenesis in vivo, which indicated that MT1 was involved in the regulation of angiogenesis [69]. Penkowa et al. observed that decreased levels of the growth factors b-fibroblast growth factor (b-FGF), transforming growth factor β1 (TGFβ1), and vascular endothelial growth factor (VEGF) could mediate the reduction in angiogenesis and regeneration in MT1+2-deficient mice after a cortical freeze injury in the central nervous system. Furthermore, the authors found that MT1+2-deficient transgenic mice expressing IL-6 displayed a dramatic reduction in IL-6-induced angiogenesis. These results suggested that MT1+2 participate in the angiogenic process possibly by regulating the expression of angiogenesis-promoting factors [70].

VEGF is a major contributor to angiogenic processes such as EC proliferation, migration, and sprouting. Wierzowiecka et al. carried out a study in three breast cancer cell lines, in which an increased expression of selected MT isoforms was induced by zinc ions to various degrees. The expression of VEGF was slightly increased after the stimulation with zinc ions, which suggested a correlation between MT expression and VEGF expression in breast cell lines [71]. Another study showed that MT3 could significantly induce the expression of VEGF through a HIF-1α-dependent mechanism in brain ECs [72]. Schuermann and colleagues also confirmed that MT2 acts upstream of VEGF expression in regulating EC proliferation, migration, and angiogenesis [73].

Matrix metallopeptidase (MMP)-9, also known as gelatinase B, is widely associated with tumor progression because of its role in extracellular matrix (ECM) remodeling, angiogenesis, and neovascularization [74]. MMP-9 is a member of the zinc-dependent metalloproteinase family that has been demonstrated to interact with MTs [75]. Hence, it can be hypothesized that MTs participate in angiogenesis via MMP-9. Zbinden et al. identified MTs as a participant in collaterogenesis and angiogenesis and observed combined dysfunction of ECs, smooth muscle cells (SMCs), and macrophages in MT knockout (KO) mice. MMP-9, platelet-derived growth factor (PDGF) receptor, and VEGF were significantly downregulated in SMCs isolated from MT KO animals, which contributed to SMC dysfunction [76].

**Fig. 3:**
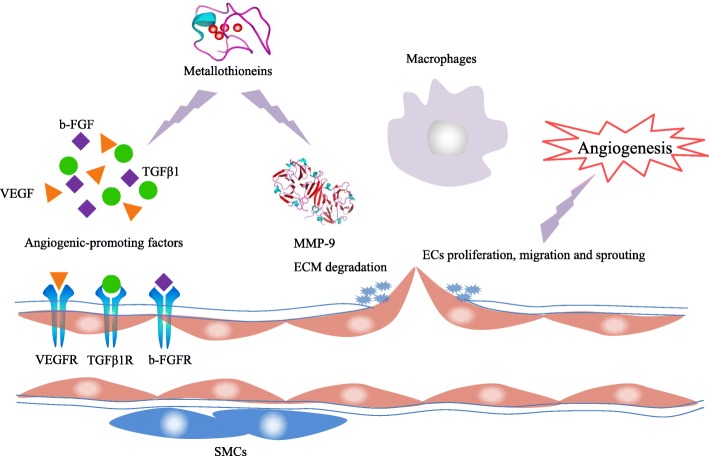
Roles of MTs in tumor angiogenesis. ECM, extracellular matrix; ECs, endothelial cells; SMCs, smooth muscle cells

In conclusion, MTs can induce the upregulation of angiogenesis-related genes, such as VEGF and MMP-9; act on ECs, SMCs, and macrophages; and result in the formation of new blood vessels to promote tumor growth, progression, and metastasis (Fig. 3). Therefore, further investigation of MTs may provide therapeutic targets for inhibiting angiogenesis and tumor progression.

#### Tumor metastasis

Metastasis is a complex, multistep process by which cancers spread from the primary site to a secondary site within the body. MT overexpression has been demonstrated as a marker of aggressive tumor behavior in many kinds of cancers. Emri et al. showed that MT1+2 overexpression was significantly more frequent in metastatic primary cutaneous malignant melanoma (CMM) (*p* = 0.018), which suggested the predictive value of MT overexpression in the metastatic ability of CMM [77]. In primary colorectal cancer, MT expression was significantly associated with lymph node metastasis, suggesting that MTs may modulate the tumor metastatic process [78]. A similar result has been reported in the lymph node metastasis of breast cancers [79]. In esophageal squamous cell carcinoma, MT expression was indicative of metastatic potential and was associated with the lymph node metastasis (*p* = 0.0343) and distant metastasis (*p* = 0.0452) [80]. In gastric cancer patients, MT overexpression was significantly correlated with lymph node and distant metastasis, as well as the number of metastatic lymph nodes [81]. Clinical studies have demonstrated that MT2A overexpression enhanced breast cancer cell invasion and migration via the upregulation of MMP-9 induced by the activation of the AP-1 and NF-κB signaling pathways [82]. In addition, studies have suggested that MT3 overexpression can increase the invasiveness of breast cancer cell by modulating MMP-3 expression [83]. Similarly, Ryu et al. verified the expression of MT1E in relation to the motility of glioma cell lines, and MT1E enhanced the invasion and migration of malignant glioma cells by modulating the activity of MMPs and NF-κB/p50 [84, 85].

Interestingly, Yan et al. conducted an in vitro study to assess the effect of MT1F expression on colon cancer cells and found that migration, invasion, and adhesion were significantly inhibited in MT1F-expressing colon cancer cells [52]. Ramaswamy et al. have shown that the downregulation of MT3 was one of the 17-gene signature associated with metastasis in primary solid tumors [86]. A decrease in MT3 expression was observed in pituitary adenocarcinoma with spinal cord metastasis [87]. Likewise, Gomulkiewicz et al. showed that the MT3 expression was significantly lower in patients with ductal breast cancer with lymph node metastasis than in patients without metastasis [88]. Furthermore, Fu et al. demonstrated a significant positive association of MT1G hypermethylation with lymph node metastasis in 178 papillary thyroid cancer patients [89].

Studies have also demonstrated that MTs are involved in tumor microenvironment remodeling to facilitate tumor spread, invasion, and metastasis [90–92].

Collectively, these data suggest that MTs contribute to tumor metastasis by enhancing the invasion and migration of tumor cells and tumor microenvironment remodeling. However, the up/downregulation of MTs depends on their isoforms and the type of tumors, as well as other environmental stimuli or gene mutations.

#### Tumor microenvironment remodeling and immune escape

Subramanian Vignesh and Deepe elucidated the immunomodulatory role of MTs and demonstrated MTs as an important component of the innate and adaptive immune systems regulating metal homeostasis, particularly zinc and thus impacting the immune cell redox status, enzyme function, and cell signaling [93]. Emri et al. observed that metastatic CMM cases were associated with the presence of tumor-infiltrating CD68+ (*p* = 0.001) and CD163+ (*p* < 0.001) macrophages. Furthermore, MT overexpression was found to be related to the presence of tumor-infiltrating CD68+ macrophages (*p* = 0.003). Hence, MTs might play an immunomodulatory role to contribute to melanoma progression [77]. MT expression has also been associated with the number and activity of immune cells during immune responses in breast cancer [94].

MTs can be released into the extracellular environment in response to cellular stress. Furthermore, extracellular MTs can bind to the plasma membrane of lymphocytes and influence their immunomodulatory activities [95]. MTs have been found to be able to suppress murine cytotoxic lymphocyte activity, reduce the level of detectable major histocompatibility complex class I and CD8 molecules on lymphocytes, and increase IL-2 receptor expression, indicating that MTs are involved in cell-mediated immunosuppression functions and contribute to antitumor immunity [96].

Studies have shown that MTs can inhibit immune responses and are involved in the microenvironment remodeling to control and accelerate tumor growth and initiate metastasis [90, 91]. Dutsch-Wicherek et al. observed that the immunoreactivity levels of MTs in pharyngeal squamous cell carcinoma were statistically higher than those in the reference tissues. In addition, higher MT immunoreactivity levels were detected in tumor patients with lymph node metastasis than in patients without metastasis. The authors concluded that MT expression within the tumor cells was associated with tumor aggressiveness and metastasis via microenvironment remodeling [91]. Canpolat and Lynes found that endogenous MTs are synthesized during the normal immune response or as a consequence of toxicant exposure suppressed in vivo immune function, indicating an immunomodulatory role of MTs [97]. Inhibition of the immune response by MTs may indicate that MTs are involved in the remodeling of the immunosuppressive tumor microenvironment [90].

These findings collectively suggest that MTs act as immunomodulators to interfere with the immune response and participate in tumor microenvironment remodeling to drive tumor immune evasion.

#### Tumor drug resistance

MTs contribute to the development of drug resistance through a variety of mechanisms in many types of cancers. A previous study has demonstrated that the overexpression of MTs was involved in the acquisition of resistance to anticancer drugs, including *cis*-diamminedichloroplatinum (II), chlorambucil, and melphalan [98]. Since then, the relationship between MT expression and tumor drug resistance has been examined in different tumor types.

In bladder carcinoma, an enhanced expression of MT2 was identified in the cisplatin-resistant cells, suggesting that cisplatin resistance may be partly mediated by MT2 [99]. This result was recently corroborated by Wülfing et al. If bladder cancer cells expressed MTs, the patients treated with cisplatin chemotherapy had a significantly poor survival rate; in other words, MT overexpression may mediate resistance to cisplatin-based chemotherapy [23]. Matsumoto et al. reported an enhanced expression of MTs following chemotherapy in non-small cell lung cancer, which may be related to drug resistance [100]. Similarly, MT expression was found to be correlated with cisplatin resistance in three human small cell lung cancer lines [101]. Lee et al. confirmed that in contrast to those in cisplatin-sensitive cells, MTs were overexpressed in cisplatin-resistant mouse melanoma cancer cells. Reducible poly(oligo-d-arginine) (rPOA) was used to deliver short hairpin RNAs against MTs (shMT) into cells and resulted in the downregulation of MT expression and enhanced the anticancer effect of cisplatin. Additionally, in vivo tumor models showed synergistically enhanced the tumor-suppressive effects of co-administration of shMT/rPOA oligopeptoplex and cisplatin. These results demonstrated that MT overexpression was at least one of the main reasons for cisplatin resistance [102]. Upregulation of MTs was shown to increase irinotecan resistance in gastric cancer patients [103]. Furthermore, MT3 overexpression increased chemotherapeutic drug resistance in PC-3 prostate cancer cells [53, 104].

Although several studies have investigated MT-mediated resistance mechanisms, the results still need to be further validated. Kondo et al. demonstrated that nuclear MTs were indicative of greater resistance to cisplatin than diffuse MTs in human hormone-independent prostatic cell lines, implying that the nuclear localization of MTs is important for the resistance to chemical drugs [105]. In addition, Surowiak et al. demonstrated that the nuclear expression of MTs increased during the exposure to cisplatin and was indicative of drug resistance in ovarian cancer cells [106]. Thus, nuclear MT expression probably represents a mechanism of drug resistance that protects the DNA of tumor cells from the toxic effects of chemical drugs. However, Gansukh et al. showed a divergent result in which the nuclear and cytoplasmic expression of MTs had no effect on cisplatin resistance in non-small cell lung cancer cells, perhaps because the mechanism of cisplatin resistance in this cancer is independent of MTs [107]. Arriaga et al. showed that the overexpression of the MT1G sensitized colorectal cancer cells to the chemotherapeutic agents’ oxaliplatin and 5-fluorouracil, which may have been mediated by the activation of p53 and repression of NF-κB activity [108]. Sun et al. also demonstrated a novel molecular mechanism of drug resistance. Specifically, they showed that enhanced MT1G expression contributed to sorafenib resistance in hepatocellular carcinoma by inhibiting lipid peroxidation-mediated ferroptosis [109]. Habel et al. showed that chemotherapy resistance induced by MT2A was partially due to zinc chelation [57]. A study by Yap et al. revealed that reduced MT2A gene expression could enhance the chemosensitivity to doxorubicin [59]. Hence, it can be speculated that inhibiting the expression of certain MT isoforms can increase the anticancer activity of drugs, thereby providing a potential therapeutic strategy.

Therefore, as a predictor of chemoresistance, MT expression might be evaluated for the selection of appropriate anticancer agents or be modulated to resensitize chemoresistant tumor cells to improve the efficacy of chemotherapy. Although MTs have an ability to increase the chemoresistance, many studies indicate that MTs can prevent cardiotoxicity induced by anticancer agents by scavenging free radicals and attenuating oxidative stress [110–112]. Hence, strategies to balance cardioprotection and chemoresistance deserve to be investigated. Heger et al. had proposed a possible solution that involved heart-specific overexpression of MTs by transient transfection to help overcome unwanted cardiotoxicity without increasing tumor chemoresistance [111]. Further studies are needed to explore the potential mechanisms of MTs, thus enhancing their cardioprotective effect while inhibiting chemoresistance.

### MTs as biomarkers for cancer diagnosis and prognosis

MTs can be readily detected in patient’s blood, and the level of serum MTs is positively correlated with the pathological state, disease stage, and degree of cancer progression; thus, MTs act as an enriched source of biomarkers.

Petrlova et al. had described various electroanalytical techniques to detect MTs in human serum [113]. Through electrochemical analysis, MT levels were found to be elevated in most patients with melanoma, breast, and colon cancer [114]. Tariba et al. selected 25 patients with newly diagnosed testicular germ cell tumors (TGCTs) and 22 healthy volunteers in their study and found that serum MT concentration in patients with TGCT was significantly higher than that in control individuals; additionally, in combination with the commonly used markers, MTs could improve the early diagnosis rate [115]. In another study with 46 prostate cancer patients diagnosed by biopsy, total prostate-specific antigen (tPSA) levels and MT levels were examined in serum samples. In the first cohort (*n* = 17) diagnosed with prostate cancer, tPSA levels were within the physiological range of 0–4 ng/mL for over 36.9% of cases, indicating the unreliability of tPSA as a marker of prostate cancer. However, although tPSA levels were normal in the first group, MT levels were significantly elevated (*p* = 0.05), indicating that MTs might be used as an additional prostate cancer marker to increase the reliability of prostate cancer diagnosis [116]. Krizkova et al. also demonstrated that determination of serum MT levels by differential pulse voltammetry could be considered as a promising diagnostic tool for childhood solid tumors [117].

Previous reports had revealed MT overexpression as a valuable prognostic marker for tumor progression and drug resistance in a wide range of cancers, such as ovarian cancer [22, 118], breast cancer [119, 120], small cell lung cancer [121], non-small cell lung cancer [122], renal cell carcinoma [123, 124], melanoma [24, 125], bladder cancer [23, 126], oral squamous cell carcinoma [127], and cholangiocarcinoma [128]. Surowiak et al. indicated that increased expression of MTs represented an unfavorable predictive factor in cisplatin-treated ovarian cancer patients [118]. In primary ovarian cancer, MT-positive patients had shorter survival than MT-negative patients, and this result could be explained by the positive correlation between MT expression and histological grade [22]. In primary invasive ductal breast carcinoma, MT-positive patients had a significantly poorer prognosis than MT-negative patients (*p* < 0.01), which suggested a prognostic value of MT expression [129]. Similarly, MT3 overexpression has been reported to be associated with poor prognosis in breast cancer [130]. The association of high MT expression with short-term survival had been demonstrated in small cell lung cancer [121] and non-small cell lung cancer [122], and enhanced expression of MT1F and MT2A isoforms predicted poor clinical outcomes in non-small cell lung cancer in which upregulated MT1F expression was associated with larger primary tumor size and higher grade of malignancy [131]. In a prospective study on 520 melanoma patients, MT overexpression was related to an increased risk of melanoma progression with poor prognosis and survival rate [125]. In 2006, the authors updated this study with an 11-year prospective cohort comprising 1270 melanoma patients to confirm the previous results [24]. Another study compared the predictive roles of MT overexpression with those of sentinel lymph node biopsy and found that MT overexpression was an excellent prognostic predictor of cancer progression and patient survival [132]. Additionally, a significant correlation was found between MT overexpression and poor overall survival (*p* = 0.0005), disease-specific survival (*p* = 0.0004), disease-free survival (*p* = 0.05), and disease-free progression (*p* = 0.0008) in patients with transitional cell carcinoma of the bladder [126]. Another study also demonstrated that the overexpression of MTs (*p* = 0.003) was an independent risk factor associated with poor survival in bladder cancer patients [23]. Similar results were observed in oral squamous cell carcinoma patients [127] and in cholangiocarcinoma patients suffering from either intrahepatic cholangiocarcinoma or hilar extrahepatic cholangiocarcinoma [128].

However, in other types of cancers such as prostate cancer [10], hepatocellular carcinoma [133, 134], and gastric cancer [135], the downregulation of MT expression was associated with poor prognosis. Demidenko and colleagues attempted to identify the prognostic biomarkers for predicting biochemical recurrence (BCR) of prostate cancer. They identified 455 differentially expressed genes through global gene expression profiling, among which seven genes (*CHI3L2*, *FABP7*, *GHRH*, *GPR52*, *MT1E*, *OLR1*, and *SAA2*) were selected for further validation in two independent prostate cancer cohorts. The results suggested that MT1E downregulation was a potential biomarker of early BCR and poor prognosis in prostate cancer patients [10]. Park and Yu demonstrated that MT1 and MT2 are important prognostic markers in hepatocellular carcinoma. The loss of nuclear expression of MT1 and MT2 was associated with high Edmondson-Steiner grade and microvascular invasion and poor prognosis indicated by recurrence-free survival (*p* = 0.029) and overall survival (*p* = 0.007) [133]. In another study, low MT1M expression was found to be linked to high alpha-fetoprotein (AFP) levels and high tumor recurrence rates following curative resection in patients with hepatocellular carcinoma [134]. In addition, combined MT1M and MT1G promoter methylation in hepatocellular carcinoma patients was associated with a high incidence of vascular invasion and lymph node or extrahepatic metastasis, thereby acting as an effective prognostic marker [136]. Decreased MT2A expression was reported to be associated with advanced TNM stages, tumor differentiation, and poor outcomes in patients with gastric cancer [135].

Taken together, these findings indicate that MTs play critical roles in almost all aspects of cancer, thereby providing opportunities for the development of MTs as novel diagnostic and prognostic biomarkers.

### Mechanisms involving MTs in cancer

MTs can act as zinc donors to mediate the activity of zinc-dependent transcription factors such as P53 and NF-κB to regulate cell apoptosis and tumor cell growth. MTs can remove zinc ions from P53 protein molecules, leading to the changes in its spatial structure and loss of function, similar to p53 mutations, and resulting in uncontrolled cell proliferation. Meplan et al. demonstrated that MT overexpression exerted a potent inhibitory effect on the transcriptional activity of P53, consistent with the metal chelation effect of MTs [137]. Interaction of MTs with the tumor suppressor P53 appears to be crucial for the development and progression of tumors [46, 138, 139]. Conversely, the activation of P53 has been shown to be an important factor in the expression and induction of MTs in cancer cells [140]. Positive correlations between MT and p53 expression were found in endometrial carcinoma, implying that MTs can regulate p53 expression [141]. The study by Hengstler et al. also showed a significant correlation between MT expression and mutant p53 in ovarian carcinoma [22]. Furthermore, the antiapoptotic role of MTs may also be related to its modulation of NF-κB activity. Abdel-Mageed and Agrawal revealed that MTs cause the transactivation of NF-κB, which results in the inhibition of apoptosis [47]. In contrast, Sakurai et al. demonstrated MTs as a negative regulator of NF-κB activity by using the MT-null embryonic cell lines [48]. MT2A has been shown to regulate NF-κB pathway activation to participate in tumor progression in gastric cancer [135] and colorectal cancer [49]. Downregulation of MT1M can also contribute to hepatocellular carcinogenesis by increasing the activity of NF-κB [142].

Numerous studies have demonstrated that the elimination of tumor suppressor gene activity by promoter methylation is responsible for carcinogenesis, a mechanism that has been confirmed in many human cancers. Demidenko et al. revealed MT1E promoter methylation as a possible mechanism of gene inactivation, which resulted in the reduced expression of MT1E in prostate cancer [10]. Similar observations were validated in patients with endometrial carcinoma [143] and melanoma [144]. In previous studies, MT1G suppression was reported to contribute to carcinogenesis in papillary thyroid carcinoma, prostate cancer, esophageal squamous cell carcinoma, hepatocellular carcinoma, and hepatoblastoma, and the mechanism of MT1G gene silencing was related to promoter hypermethylation [145–150]. In gastric carcinoma, Deng et al. showed that the reduced expression of MT3 was due to the hypermethylation of CpG islands on intron 1 [151]. DNA methylation of MT3 was detected in esophageal adenocarcinoma, resulting in MT3 gene silencing. Moreover, DNA methylation of MT3 from − 127 to − 8 sites was shown to be significantly correlated with advanced tumor stages and lymph node metastasis, implying that the methylation of promoter regions may be involved in tumor progression [152]. DNA methylation of MT3 was also confirmed in esophageal squamous cell carcinoma, but there was no significant association between the MT3 methylation status and prognosis [153]. Tao et al. observed epigenetic inactivation of MT3 via promoter hypermethylation in pediatric acute myeloid leukemia, and MT3 could act as a tumor suppressor by inhibiting tumor cell proliferation and inducing apoptosis [154]. Han et al. demonstrated that MT1H acts as a tumor suppressor by interacting with euchromatin histone methyltransferase 1 (EHMT1) to increase the methyltransferase activity of EHMT1 on histone 3 [26]. Earlier studies have shown that MT1G inactivation was mediated by promoter methylation in thyroid cancer [7, 149]. Fu et al. revealed in-depth molecular mechanisms of MT1G as a tumor suppressor in thyroid carcinogenesis, which involved the inhibition of cell growth and invasion and induction of cell cycle arrest and apoptosis via the inhibition of the phosphorylation of Akt and Rb, that is, through modulation of the phosphatidylinositol 3-kinase (PI3K)/AKT and Rb/E2F signaling pathways [89].

Beyond DNA methylation, there are other potential mechanisms of MTs in carcinogenesis. The suppression of MT1 and MT2A in human hepatocellular carcinoma was related to the dephosphorylation (inactivation) of the transcription factor CCAAT/enhancer-binding protein (C/EBP) α through the PI3K/AKT signaling pathway rather than the gene hypermethylation [25]. Zheng et al. established stable hepatocellular cancer cell lines with constitutive expression of MT1H to examine the potential role of MT1H in hepatocellular carcinogenesis. They found that MT1H could suppress Wnt/β-catenin signaling to inhibit tumor progression, including hepatocellular cancer cell proliferation, invasion, and migration [8]. In terms of the studies on genomic changes, Chan et al. noted that MT1G downregulation in hepatocellular carcinoma was due to the allelic loss on chromosome 16q12.1-q23.1 [155]. Additionally, Yan et al. demonstrated that the potential mechanism of MT1F downregulation in colon cancer was a loss of heterozygosity (LOH) [52]. Furthermore, Krzeslak et al. found that single nucleotide polymorphism (SNP) (rs28336003) could affect the expression of MT2A in prostate cancer and that this may be associated with metal accumulation, causing the cells to lose protection against heavy metal toxicity and carcinogenicity [14].

As described above, many potential molecular mechanisms of MTs in tumorigenesis have been reported by scholars (Table 3), but countless challenges remain to be overcome. Further studies should be conducted to fully elucidate the exact mechanisms mediating the complex roles of MTs in cancer. In addition to focusing on currently known mechanisms, we need to do a search for potential targets and ultimately provide novel strategies for cancer therapy.

**Table 3:** Roles of MTs in carcinogenesis

MT isoforms	Mechanisms	References
MTs	Regulation of P53 activity	[22, 46, 137–139, 141]
MTs	Regulation of NF-κB activity	[47–49, 135, 142]
MTs	Methylation of DNA promoters	[7, 10, 143–152, 154, 204]
MT1H	Increasing the methyltransferase activity of EHMT1 on histone 3	[26]
MT1G	Modulation of the PI3K/AKT and Rb/E2F signaling pathways	[89]
MT1, MT2A	Modulation of the PI3K/AKT signaling pathway	[25]
MT1H	Suppression of the Wnt/β-catenin signaling pathway	[8]
MT1G	Allelic loss on chromosome 16q12.1-q23.1	[155]
MT1F	Loss of heterozygosity	[52]
MT2A	SNP (rs28336003)	[14]

### MTs as cancer therapeutic targets

Given the importance of MTs in physiological and pathological processes, accumulating data have exhibited the possible role of MTs as a therapeutic molecular target against human diseases, such as neurodegenerative diseases, cerebral ischemia and retinal diseases, liver diseases, chemical- and radiation-induced carcinogenesis, pulmonary inflammation, and obesity [156–158].

MT overexpression promotes cell growth, angiogenesis, metastasis, and chemoresistance in many kinds of human tumors. Thus, MT gene KO is gaining interest as a therapeutic approach for the treatment of these cancers. RNA interference is widely employed as a strategy to stably inhibit gene expression. Packaging RNA (pRNA) of bacteriophage phi29 has been used to deliver short interfering RNA (siRNA) into the cells for gene therapy [159, 160]. Tarapore et al. constructed pRNA/siRNA chimeras targeting MT2A in ovarian cancer cells and showed that the pRNA/siRNA complex could inhibit the expression of MT2A, resulting in a decreased cell proliferation. This result was also confirmed in breast and prostate cancer cells [161]. Therefore, the pRNA/siMT2A chimera represents a highly potent therapeutic approach against cancer. Lai et al. reported that silencing the MT2A gene by siRNAs induced entosis (the internalization of a cell into another cell) [162] in breast cancer, and this result may provide new insights into strategies to limit tumor cell growth [163]. Similarly, Lee et al. used shMT/rPOA oligopeptoplexes to downregulate MT expression and reverse the cisplatin resistance and verified enhanced anticancer efficacy in both cisplatin-resistant cell lines and in vivo mouse cancer models [102]. Antisense approaches targeting unique mRNA molecules are intended to reduce the expression of specific proteins, and this strategy is possibly applicable for cancer therapy [164]. The downregulation of MTs by antisense approaches has been shown to induce growth inhibition in breast cancer cells [165], leukemia cells [166], and nasopharyngeal cancer [167].

Sharma et al. highlighted the clinical significance of MTs in cell therapy and nanomedicine [168]. The study and application of nanoparticles (NPs) are increasingly gaining focus in biological systems and nanomedicine. MT expression can be specifically induced by metal nanoparticles and cancer to serve as a defensive mechanism and provide protection by acting as free radical scavengers, anti-inflammatory agents, and antiapoptotic agents and by mediating zinc-dependent gene expression involved in cell apoptosis, proliferation, or differentiation [111, 168, 169]. Hence, MTs could be used as early and sensitive biomarkers to assess the effectiveness and environmental safety of newly developed NPs. Based on these properties, MT-capped semiconductor NPs are now being further developed for their theranostic applications as third-generation NPs.

MT isoforms can be targeted to treat cancer and enhance the efficacy of anticancer therapies. However, not many strategies of targeting MT isoforms have reached the clinical practice as therapeutic agents. Further work is needed to achieve and update these technologies and to evaluate the clinical safety of these strategies modulating MT expression. Targeting particular MT isoforms in various cancer types indicates a promising future for the biomedical applications of MTs in the field of cancer treatment.

## Conclusions

MTs, a unique class of metalloproteins, are emerging as important players in carcinogenesis. MTs play a pivotal role in multiple biological processes by virtue of their unusual metal-binding functions, such as participating in metal ion homeostasis and detoxification, regulating cell growth and proliferation, and protecting the body against DNA damage and oxidative stress. Moreover, currently available experimental evidence suggests vital roles of MTs in tumor growth, differentiation, angiogenesis, metastasis, immune escape, and drug resistance. However, the data on the relationship between MT expression and tumor types are variable, that is, MT expression is not universal in all human cancers not only because the functions of MTs are isoform- and tissue-specific but also because MT expression varies with different environmental stimuli or gene mutations and interactions with other cell signaling pathways or the tumor microenvironment. In addition, contradictory results in the same kind of cancer may be because some studies had only been carried out in a small number of cases. This review provides a comprehensive summary of the complicated roles of MTs in carcinogenesis. The identification of changes in the expression of particular MT isoforms can contribute to tumor diagnosis and targeted therapy. Future studies of MTs will not only reveal their functions in the pathogenesis of cancer but also provide new insights into cancer diagnosis and therapy.

## Abbreviations

AFP: Alpha-fetoprotein
ATM: Ataxia telangiectasia mutated
ATRA: All-trans retinoic acid
BCR: Biochemical recurrence
b-FGF: b-fibroblast growth factor
C/EBP: CCAAT/enhancer-binding protein
cdc25A: Cell division cycle 25A
CMM: Cutaneous malignant melanoma
ECM: Extracellular matrix
ECs: Endothelial cells
EHMT1: Euchromatin histone methyltransferase 1
ELFEMFs: Extremely low-frequency electromagnetic fields
ER: Estrogen receptor
FADD: Fas-associated death domain
GIF: Growth inhibitory factor
HIF-1α: Hypoxia-inducible factor-1α
IL: Interleukin
KO: Knockout
LOH: Loss of heterozygosity
MCM-2: Minichromosome maintenance protein 2
MMP: Matrix metalloproteinase
MTs: Metallothioneins
NCBI: National Center of Biotechnology Information
NF-κB: Nuclear factor-κB
NPs: Nanoparticles
PDGF: Platelet-derived growth factor
PI3K: Phosphatidylinositol 3-kinase
pRNA: Packaging RNA
rPOA: Reducible poly(oligo-d-arginine)
shMT: Short hairpin RNAs against MTs
siRNA: Short interfering RNA
SMCs: Smooth muscle cells
SNP: Single nucleotide polymorphism
TGCTs: Testicular germ cell tumors
TGFβ1: Transforming growth factor β1
TNM: Tumor node metastasis
tPSA: Total prostate-specific antigen
VEGF: Vascular endothelial growth factor
